# The use of an electronic form to register play observation of a child with anxiety: A study case at a university clinical practice in Brazil

**DOI:** 10.1192/j.eurpsy.2021.602

**Published:** 2021-08-13

**Authors:** G. Silva, J. Soares, T. Brandão, C. Varanda

**Affiliations:** Institute Of Human Sciences - Faculty Of Psychology, Universidade Paulista, SANTOS, Brazil

**Keywords:** Treatment

## Abstract

**Introduction:**

The modality of assessment used at a University Clinical Practice in Brazil is interventive psychodiagnosis in which the active participation of children and families is considered. Orientation is given following the input provided by children and their parents.

**Objectives:**

Evaluating the use of an electronic form to be fulfilled during the observation of a child’s play in psychological session.

**Methods:**

A child at the age of 5yrs 4m was brought for psychological assessment with the complaint of aggressiveness and irritability. His parents answered the Child Behavior Checklist (CBCL -1 1/12 5 yrs) and the Psychology interns had to observe the child’s play and fulfill an electronic form in which the choice of toys and plays, motricity, creativity, symbolic abilities, frustration tolerance, adequation with reality were verified.

**Results:**

The results of CBCL indicated that the child was within the clinical range regarding anxiety and depression along with somatic complaints. The indicators observed in the electronic form such as rigidity in the modality of play, the lack of adequate ability of impersonating in role-playing, the difficulty of using creativity during play unless he was guided by peers or the Psychology interns and the constant anguish of separating himself from his parents were crucial for parents’ orientation. The psychological treatment lasted five months and benefited from the information obtained through the form once the symptoms of irritability and aggressiveness were reduced.

**Conclusions:**

This modality of assessment can be instructional for parents and may also reduce financial and time costs once provides specific indicators to observe during play.

